# Metabolic syndrome and the early detection of impaired glucose tolerance among professionals living in Beijing, China: a cross sectional study

**DOI:** 10.1186/1758-5996-5-65

**Published:** 2013-11-06

**Authors:** Ping Zeng, Xuefeng Zhu, Yi Zhang, Sinan Wu, Jun Dong, Tiemei Zhang, Shu Wang

**Affiliations:** 1 The Key Laboratory of Geriatrics, Beijing Hospital & Beijing Institute of Geriatrics, Ministry of Health, Beijing 100730, China; 2 Department of Outpatient, Chinese Academy of Sciences, Beijing 100864, China

**Keywords:** Metabolic syndrome, Impaired glucose tolerance, Impaired fasting glucose

## Abstract

**Background:**

The purpose of this study is to investigate the association of metabolic syndrome (MS) and its components with the risk of impaired glucose tolerance (IGT) in high risk urban professionals. The goal is to improve the selection of candidates who would most benefit from an oral glucose tolerance test (OGTT).

**Methods:**

This is a cross sectional study in which MS was identified by both the definitions proposed by the National Cholesterol Education Program (NCEP) and the International Diabetes Federation (IDF).

**Results:**

There were 928 eligible subjects in the study, and 23.9% of them failed in OGTT. The odds ratio of IGT was increased 3.16-fold for MS defined by the NCEP criteria and 2.79-fold for the hyperglycemia factor alone. Both MS and hyperglycemia were shown to be acceptable measures to discriminate subjects with IGT from those with normal glucose tolerance (NGT). The clustering of any 1, 2, or ≥3 metabolic components resulted in increased odds ratios for IGT: i.e., 1.71, 2.38 and 5.92, respectively. Even without hyperglycemia in the cluster, an increased odds ratio was still observed. The risk of IGT increased dramatically when the fasting plasma glucose and waist circumference were both at their highest defined level.

**Conclusions:**

MS and its components are associated with the increased risk of IGT. People with MS, one of its components, especially hyperglycemia and central obesity, or a cluster of its components are strong candidates for an OGTT in order to achieve early cost-effective detection of IGT.

## Background

The prevalence of diabetes is rapidly increasing in China and throughout the world [1-3]. Meanwhile, diabetes often remains undiagnosed for a long period of time, during which irreversible damage to multiple organs can occur [4-9]. Although evidence is clear that early detection and intervention of diabetes play important roles in diabetes control [10,11], there are still many challenges in developing effective methods to identify high risk individuals as early as possible [4-6,12-16]. Of all the screening tests, a fasting glucose test is a comparatively simple and pragmatic approach to assess the glycemic status of an individual. However, there is increasing evidence that fasting glucose alone misses many persons who might fail the more rigorous oral glucose tolerance test (OGTT) [7-9,13-16], which is required to identify patients with impaired glucose tolerance (IGT). Most importantly, IGT has been revealed to be a stronger predictor of future diabetes than impaired fasting glucose (IFG) [16-18]. Once these patients are identified, lifestyle modification and medication have been shown to prevent or delay the progression to diabetes [10,11]. However, the disadvantages of OGTT -- its inconvenience and cost -- limit its use for large-scale screening.

Metabolic syndrome (MS) has been proposed as pre-diabetic status in various ethnic groups, and many studies have confirmed the association between MS and the subsequent development of diabetes [19-27]. In contrast, fewer studies have investigated the association between MS and the risk of IGT [4,6,27-29]. When compared to OGTT, the components of MS -- measures of waist circumference (WC), blood pressure (BP), fasting plasma glucose (FPG), high-density-lipoprotein-cholesterol (HDL-C) and triglycerides (TG) levels -- are far more widely gathered during routine checkups. Therefore, given these circumstances, it is useful to evaluate the association between MS and IGT cross-sectionally, in order to improve the selection of candidates who would most benefit from an OGTT. In China, however, the probability of IGT given MS is usually underestimated by clinicians when evaluating patients with normal fasting glucose levels.

In this study, we assess the association of MS, as defined by both the updated US National Cholesterol Education Program (NCEP) Adult Treatment Panel III (ATP III) [30] and the International Diabetes Federation (IDF) [31], with the probability of IGT in a group of professionals in Beijing, China. We used both sets of criteria because the IDF definition requires abdominal obesity as the pre-condition for the diagnosis of MS. In contrast, the NCEP criteria do not have this requirement, and therefore it is more inclusive in the diagnosis of MS. We also analyzed the associations of individual components and their clustering with the risk of IGT in this population.

## Methods

### Data collections

The study, approved by the Ethics Committee of Beijing Hospital, Ministry of Health, was carried out in two research institutes in Beijing. The interviewers were trained before the survey was administered. The questionnaire was designed to collect information on demographics, lifestyle, history of diseases, and physical and laboratory examination findings. Self-reported history of type 2 diabetes (ever-diagnosed or currently taking medicine to control glucose level), hypertension (ever-diagnosed, or taking medicine to control blood pressure), and family history of diabetes and hypertension were assessed. When the waist circumference (WC) was measured for central obesity, the subject stood, and the measurement was made at the level of umbilicus at the end of normal expiration. Blood pressures (BP) were measured twice in a sitting position, and the mean values were used in the assessment. Blood samples were taken after a fast of at least 12 hours and were measured in the clinical laboratory of Beijing Hospital, where the analyses were calibrated by Cholesterol Reference Method Laboratory Network (CRMLN) Member Laboratory (The Key Laboratory of Geriatrics, Beijing Hospital & Beijing Institute of Geriatrics, Ministry of Health).

### Definitions

After finishing their regular health examinations, the volunteers consumed a standardized 75-g glucose load, and glucose levels were measured again 2 hours later. IGT is defined as glucose level at 2 hours post-glucose loading (2 hPG) from ≥7.8 mmol/l to <11.1 mmol/l [32]. Impaired fasting glucose (IFG) is defined as FPG levels of 6.1-6.9 mmol/l [32].

The criteria for the components of MS were defined as: 1. Central obesity: WC ≥90 cm for men and ≥80 cm for women; 2. High triglyceride level: TG ≥1.70 mmol/l (150 mg/dl), or under specific treatment for this lipid abnormality; 3. Low high-density-lipoprotein-cholesterol: HDL-C <1.03 mmol/l (40 mg/dl) for men and <1.29 mmol/l (50 mg/dl) for women or under specific treatment for this lipid abnormality; 4. Elevated blood pressure: systolic blood pressure (SBP) ≥130 mmHg, or diastolic blood pressure (DBP) ≥85 mmHg, or having previously diagnosed hypertension; 5. Hyperglycemia: fasting plasma glucose (FPG) ≥5.6 mmol/l (100 mg/dl). The IDF criteria for MS are central obesity plus ≥2 of the other four factors. The NCEP ATP-III criteria are similar, except central obesity is not a prerequisite, being defined simply as any three of the five sub-optimal conditions above.

### Subjects

In this study, persons aged ≥20 were invited to participate in this study when they took their annual health examinations. There were 1242 informed consent volunteers enrolled in the study, of whom 65% performed office work, such as research; 7% worked in maintenance and other areas; and 28% were retired. People with known IGT or diabetes, or who were taking medication for these conditions, or whose FPG levels were ≥7.0 mol/l, or whose 2 hPG ≥11.1 mmol/l in this examination were not included in the study, leaving a total of 928 subjects.

### Statistical analysis

The different characteristics of subjects in the group with normal glucose tolerance (NGT) (2 hPG <7.8 mmol/l) and those in the group with IGT were compared using one-way ANOVA tests and Chi-square tests. Logistic regression was used in multivariable models to estimate the adjusted odds ratios and 95% confidence interval (95% CI). The area under the receiver-operating characteristic curves (AROCs) was used to estimate the ability of MS or its components to discriminate subjects with IGT from those without IGT. The higher the AROC, the better the discrimination. Attributable risk percentage (AR%) is the excess risk of IGT attributable to MS or its components. Population attributable risk percentage (PAR%) is defined as the excess rate of IGT in a population associated with MS or its components. When the relationships between the numbers of abnormalities and IGT were assessed, the group without any disorders was set as the reference group. The dummy variables were created for persons with 1, 2, ≥3 metabolic components. The statistical analyses were carried out using SAS software licensed to Chinese Center for Disease Control and Prevention, and *p* <0.05 was considered statistically significant.

## Results

### The characteristics of the study subjects

The different characteristics of subjects with NGT (2 hPG < 7.8 mmol/l) and IGT are shown in Table 1. There were a total of 928 eligible subjects (403 males and 525 females) without diagnosed diabetes who participated in this study. 222 subjects (85 males and 137 females) were found to have IGT. Persons with MS defined by both IDF criteria and NCEP criteria had statistically higher risk of presenting IGT than its reference group (that is, those not meeting either the IDF or NCEP criteria of MS): 58.1% vs. 25.5% for NCEP criteria, and 52.7% vs. 22.8% for IDF criteria. Also, the subjects with IGT tended to be older and have higher values for WC, FPG, TG, weight, BMI, systolic BP, diastolic BP and lower HDL cholesterol. Unexpectedly, the risk of IGT was not related to family history of diabetes nor educational attainment less than 12 years.

**Table 1:** **Characterization of study subjects with normal glucose tolerance (NGT) and impaired glucose tolerance (IGT)**^**a**^

	**NGT**^**b**^	**IGT**^**c**^	***p*-value**
	**(n = 706)**	**(n = 222)**	
Gender			0.0767
Male (n = 403)	45.0	38.3	
Female (n = 525)	55.0	61.7	
Age	45 ± 15	57 ± 15	<0.0001
Waist circumference (cm)	83 ± 10	89 ± 9	<0.0001
Fasting glucose level (mmol/l)	5.17 ± 0.51	5.57 ± 0.52	<0.0001
2-h Post loading glucose level (mmol/l)	6.15 ± 0.95	8.88 ± 0.92	<0.0001
Triglyceride (mmol/l)	1.50 ± 0.96	1.84 ± 1.02	<0.0001
HDL cholesterol (mmol/l)	1.34 ± 0.34	1.30 ± 0.33	0.1423
Weight (kg)	66 ± 13	67 ± 13	0.3205
Body mass index (kg/m^2^)	23.6 ± 3.6	24.8 ± 3.7	<0.0001
Systolic blood pressure (mmHg)	117 ± 15	126 ± 16	<0.0001
Diastolic blood pressure (mmHg)	78 ± 9	81 ± 10	0.0002
Family History of Diabetes	23.9	23.0	0.0868
Education (<12 years)	7.5	8.1	0.7689
MS NCEP criteria	25.5	58.1	<0.0001
MS IDF criteria	22.8	52.7	<0.0001

^a^Data are mean ± SD, or n(%). HDL: high density lipoprotein; MS: metabolic syndrome; NCEP: National Cholesterol Education Program; IDF: International Diabetes Federation. ^b^NGT: glucose level at 2 hours post-glucose loading (2 hPG) <7.8 mmol/l; ^c^IGT: impaired glucose tolerance.

### The association of impaired glucose tolerance with metabolic syndrome and its components

The association of IGT with MS and individual components of MS are shown in Table 2. Central obesity presented the highest prevalence rate (52.7%) and followed by high blood pressure (44.7%) in this population. About 30% of the population displayed MS or any one of the other metabolic components. The odds ratio of IGT were increased 3.16-fold for MS, defined by the NCEP criteria, 2.84-fold for MS, defined by the IDF criteria, and 2.79-fold for the hyperglycemia factor, and all of them had greater than 60% probability to discriminate persons with IGT from persons without IGT. For AR%, the risk of IGT was over 60% attributable to both MS (by either definition) and hyperglycemia. For PAR%, about one third of IGT in the population might be attributable to MS (by either definition), hyperglycemia, central obesity and high blood pressure. In contrast, high TG and low HDL-C each had a lower association with IGT.

**Table 2:** **Association of IGT with the components of metabolic syndrome**^**a**^

	**Prevalence (%)**	**Number of IGT (%)**	**OR (95% CI)**	**AROC (95% CI)**	**AR %**	**PAR %**
MS-NCEP	309 (33.3)	129 (41.8)	3.16 (2.26-4.42)	63.4 (60.3-66.5)	64.0	37.2
MS-IDF	278 (30.0)	132 (42.1)	2.84 (2.03-3.97)	63.0 (60.0 -66.0)	61.6	32.5
Central obesity	489 (52.7)	158 (32.3)	2.06 (1.46-2.91)	58.9 (56.2-61.5)	54.9	39.1
Hyperglycemia	279 (30.1)	118 (42.3)	2.79 (1.99-3.91)	66.9 (61.5-72.3)	61.3	32.6
High TG	293 (31.6)	96 (32.8)	1.95 (1.39-2.74)	56.5 (53.4-59.6)	39.4	17.1
Low HDL-C	301 (32.4)	91 (30.2)	1.65 (1.19-2.30)	54.7 (51.6-57.7)	30.9	12.7
High BP	415 (44.7)	141 (34.0)	1.61 (1.13-2.30)	59.1 (56.3-62.9)	53.5	34.0

^a^Logistic regression was age-and gender-adjusted. IGT: impaired glucose tolerance; MS: metabolic syndrome; NCEP: National Cholesterol Education Program; IDF: International Diabetes Federation; TG: triglycerides; HDL-C: high-density-lipoprotein cholesterol; BP: blood pressure; AROC: receiver-operating characteristic curve; AR %: Attributable risk percentage; PAR %: Population attributable risk percentage.

### The association of impaired glucose tolerance with the clustering of metabolic components

The risk associations of the clustering of metabolic components are shown in Table 3. The risk of IGT increased as the number of metabolic components included in the cluster increased. Although OR decreased a little when hyperglycemia was excluded from the number of components in the clustering, a rising risk gradient was still observed. When the combinations of metabolic components were analyzed, the clusters that included hyperglycemia exhibited the highest association with IGT. When hyperglycemia was excluded from the clustering, the combinations with central obesity showed the greatest increased risk of IGT.

**Table 3:** The effect of clustering of metabolic syndrome components on the risk of IGT, determined by logistic regression

	**Number of clustering**	**Prevalence n (%)**	**Number of IGT (%)**	**OR (95% CI)**	**The combination of metabolic components in the cluster with the most prevalent rate of IGT**
Including hyperglycemia	0	189 (20.4)	13 (6.9)	Ref	
	1	200 (21.6)	29 (14.5)	1.71 (0.85-3.46)	hyperglycemia
	2	230 (24.8)	51 (22.2)	2.38 (1.22-4.67)	hyperglycemia + high BP
	≥3	309 (33.2)	129 (41.7)	5.92 (3.12-11.21)	hyperglycemia + high BP + central obesity
Without hyperglycemia					
	1	178 (27.1)	24 (13.4)	1.53 (0.74-3.19)	Central obesity
	2	169 (25.8)	35 (20.6)	2.21 (1.08-4.53)	Central obesity + high BP
	≥3	120 (18.3)	38 (31.7)	4.44 (2.14-9.20)	Central obesity + high BP + high TG

Logistic regression was age-and gender-adjusted. IGT; impaired glucose tolerance; BP: blood pressure; TG: triglyceride.

### The effect of combining central obesity and hyperglycemia on the risk of impaired glucose tolerance

The effect of combining WC and FPG on the risk of IGT is shown in Table 4. The IGT risk increased with increasing waist circumference and/or increasing fasting glucose level. The risk of IGT increased dramatically when both FPG and WC were present at their highest levels.

**Table 4:** **The combined effect of waist circumference level and fasting glucose level on the risk of IGT**^**a**^

**Waist circumference level**	**FPG <5.6 mmol/l (n = 657)**		**FPG: 5.6-6.0 mmol/l (n = 183)**		**FPG ≥6.1 mmol/l (n = 88)**	
	**n1**	**Prevalence of IGT (%)**	**n2**	**Prevalence of IGT (%)**	**n3**	**Prevalence of IGT (%)**
1	350	34 (9.7)	66	20 (31.3)	23	12 (43.5)
2	218	52 (23.9)	77	26 (33.8)	33	19 (57.6)
3	89	23 (25.8)	40	19 (47.5)	32	22 (59.4)

^a^Trend P < 0.0001. IGT: impaired glucose tolerance; FPG: fasting plasma glucose. Waist circumference (WC) level: 1: WC <90 cm for men and <80 cm for women; 2: WC ≥90 cm but <102 cm for men, and ≥80 cm but <88 cm for women; 3: WC ≥102 cm for men and ≥88 cm for women.

## Discussion

Type 2 diabetes has become a significant human health disease threat across the globe. Although early detection and intervention are recognized as the most important and effective way to prevent or delay the onset of diabetes or its complications [7-11], data from the year 2000 showed that three out of four diabetes patients in China were undiagnosed [2], and data from 2007 showed that three out of five diabetes patients in China remained undiagnosed [1]. To some extent, these findings can be explained by the low rate of regular checkups in general population in China. In our study, the subjects could easily access regular health examination, and their fasting glucose levels were checked annually. However, 23.9% of individuals still failed the two-hour post challenge glucose test. In addition, previous studies have observed about 35-39% of IGT and 22-31% of diabetes was undiagnosed in acute heart attack patients [7-9]. Taken together, these results support the importance of increasing the early detection of IGT in the population.

MS has been widely accepted as a predictor of future diabetes [19-27]. However, the cross-sectional association of MS with IGT has not been widely evaluated in the Chinese population. In our study, MS was associated with about a 3-fold increase in the risk of IGT. In the study of Meigs et al., similar results were also observed among their study population, which included Caucasians, Mexican-Americans, and African-Americans, where ORs for IGT of 3–4 were observed [29]. Further, MS has acceptable power to discriminate subjects with IGT from those without IGT (AROC about 63%) in this population. The prevalence of MS defined by the NCEP criteria is higher than that defined by the IDF criteria (33.3% vs. 30.0%) in our study subjects, since central obesity is a prerequisite for the IDF definition [30,31]. OR, AROC and AR% and PAR% are also somewhat higher for the NCEP criteria than for the IDF criteria (i.e., 3.16, 63.4%, 64.0%, 37.2%, respectively, vs. 2.84, 63.0%, 61.6%, 32.5%, respectively, as shown in Table 2), but the magnitudes are quite similar. In general, MS, whether defined by the NCEP or IDF criteria, is useful for identifying OGTT candidates.

Among the single components of MS, hyperglycemia showed the highest association with IGT with OR (95%CI) being 2.79 (1.99-3.91), which is very close to MS defined by the IDF criteria (OR = 2.84, 95% CI: 2.03-3.79). In our previous study in a cohort of 7922 subjects, a much higher association of hyperglycemia with the future development of diabetes was observed (OR about 5.6 for MS vs. 9.1 for hyperglycemia) [19]. Many other longitudinal studies, such as Hongkong Study [24] and the Framingham Offspring Study [25] also support the notion that hyperglycemia is more predictive than MS. The similar association of hyperglycemia and MS with IGT in this cross sectional study can be at least partially explained by the strong association of IGT with insulin resistance [33,34], which is the underlying pathophysiology of MS [35].

IDF, NCEP and American Diabetes Association (ADA) define hyperglycemia at a fasting glucose level of ≥5.6 mmol/l, and therefore more individuals with this MS component can be identified [30-32]. However, since the prevalence of hyperglycemia is lower than that of MS or the other components in the general population, especially in Chinese [1,2,36], using fasting glucose alone is insufficient to identify most IGT individuals [13-16]. At the same time, it is important to recognize that the metabolic mechanisms that underlie hyperglycemia and IGT are somewhat different. Specifically, hyperglycemia is characterized by elevated hepatic glucose output and a defect in early insulin secretion, while IGT is characterized by peripheral insulin resistance. Therefore, there is limited overlap between the two factors, and they define different groups of subjects [37].

Many studies have shown that forming clusters with more metabolic variables than hyperglycemia alone increases the prediction of diabetes/IGT greatly [4,6,17,19,20,25]. In this study, we observed that clustering of any 1, 2, and ≥3 metabolic components gradually increases the association with the risk of IGT, with OR (95% CI) being 1.71 (0.85-3.46), 2.38 (1.22-4.67), and 5.92 (3.12-11.21), respectively. Notably, the increased IGT risk was still observed when hyperglycemia was excluded from the clustering of metabolic components. Similar findings have already been reported in other prospective studies, including ours, which analyzed the association of MS and its components with the future development of diabetes [19,20,25].

Central obesity has been suggested as an important risk factor of insulin resistance [38], and central obesity has been widely regarded as an important factor in estimating the risk of IGT [19,24,25,29]. In this study, central obesity is confirmed to have the ability to identify IGT subjects, with an OR (95% CI) being 2.06 (1.46-2.91), which is second highest only to hyperglycemia. Similar results have also been observed in cohort studies [19,24]. In this current study, the pattern of interactions between waist circumference (WC) level and FPG level were revealed. According to the central obesity criteria defined for Asians and Westerners [30,31], WC were classified into 3 levels: 1, waist circumference <90 cm for men and < 80 cm for women; 2, waist circumference 90–101 cm for men, and 80–87 cm for women; 3, waist circumference ≥102 cm for men and ≥88 cm for women, respectively. FPG levels were defined as: FPG <5.6 mmol/l, FPG = 5.6-6.0 mmol/l, and FPG ≥6.1 mmol/l (see Table 4). The association with IGT is stronger with FPG level than with WC level. Given a WC level that remained unchanged, the risk of IGT increased about 15-35% as the level of FPG increased. Given a FPG level that remained unchanged, the risk of IGT increased about 7-18% as the level of WC increased. Clustering these two metabolic components increased the risk even further. This increased risk of IGT was dramatic when the two variables were both present at their highest defined levels. Although the criteria for the definition of central obesity are lower in the Asians than in the Westerners [30,31], persons with hyperglycemia and at the higher level of WC, deserve close scrutiny.

The prevalence of high BP in this population was as high as 44.7%, and a significant association with IGT was observed (see Table 2, OR = 1.61, AROC % = 59.1, AR % = 53.5, PAR % = 34.0). The high prevalence of high BP may help explain the results in Table 3 which shows that high BP combined with hyperglycemia in the metabolic clustering to exhibit the highest association with IGT among clusters of the same number of components. Since the Chinese population is vulnerable to high BP [36], these results arouse special concerns when evaluating the risk of IGT.

It must be mentioned that our study has a few limitation. First, the prevalence of MS in this population was much higher than that observed in our previous study (about 20%) and in the general population (9.8% for men and 17.8% for women) [19,36]. The high prevalence of MS in this study is consistent with the hypothesis that this population would be at high risk of MS and diabetes due to their sedentary working style and high work-related stress [19,36]. Moreover, health support or advice has raised awareness of diabetic risk in the population and may have made more obesity persons willing to volunteer to participate in this study. Consequently, the results are likely to be less representative of the general population. However, our findings are very informative for the high risk individuals, such as those in this study, to realize the risk of diabetes if they already have metabolic syndrome or the clustering of its components.

Second, our sample size is not big enough to divide subjects into groups according to age and gender. Thus the age- and gender- specific association between factors and the risk of IGT were not revealed. However, the purpose of this study focuses on whether MS is an effective method for helping to identify high risk individuals as early as possible. Based on the findings from this study, it is confirmed that persons with MS are very likely to fail OGTT, and IGT can be detected in subjects who have normal FPG. Furthermore, we found over 60% of the excess risk of IGT attributable to MS, and about 32-37% of IGT in the population attributable to MS. These data suggest that an effective strategy to reduce IGT involves intensive intervention to return MS components back to normal.

## Conclusion

In summary, this study shows that metabolic syndrome (MS) and its components can be used to evaluate the risk status of IGT in the growing population of urban professionals in China. People with MS or its components, especially hyperglycemia, central obesity, or with the cluster of its components are strong candidates for an oral glucose tolerance test in order to achieve early detection of IGT. This information should be publicized in the target population and the medical professionals who serve this important human resource in China. Further research will seek to refine these data to examine age and gender related issues.

## Abbreviations

MS: Metabolic syndrome; NCEP: National cholesterol education program; IDF: International diabetes federation; OGTT: Oral glucose tolerance test; IFG: Impaired fasting glucose; IGT: Impaired glucose tolerance; 2hPG: 2 hours post-challenge glucose level; NGT: Normal glucose tolerance with 2 hPG < 7.8 mmol/l; FPG: Fasting plasma glucose; HDL-C: High-density-lipoprotein cholesterol; TG: Triglycerides; BP: Blood pressure; WC: Waist circumference; BMI: Body mass index; AROCs: Receiver-operating characteristic curves; AR %: Attributable risk percentage; PAR %: Population attributable risk percentage.

## Competing interests

The authors declare that they have no competing interests.

## Authors’ contributions

PZ designed the study, conducted the analyses, and wrote the manuscript. XFZ, YZ, SNW, and JD made contributions to the conception and design of the study, the acquisition of data and review the manuscript. TMZ and SW contributed to the design of the study, interpretation of data, and revising the article critically for important intellectual content. All authors read and approved the final manuscript.
